# A Novel *In Vivo* Vascular Imaging Approach for Hierarchical Quantification of Vasculature Using Contrast Enhanced Micro-Computed Tomography

**DOI:** 10.1371/journal.pone.0086562

**Published:** 2014-01-27

**Authors:** Laura Nebuloni, Gisela A. Kuhn, Johannes Vogel, Ralph Müller

**Affiliations:** 1 Institute for Biomechanics, ETH Zurich, Zurich, Switzerland; 2 Institute of Veterinary Physiology and Zurich Center for Integrative Human Physiology, University of Zurich, Zurich, Switzerland; Mayo Clinic College of Medicine, United States of America

## Abstract

The vasculature of body tissues is continuously subject to remodeling processes originating at the micro-vascular level. The formation of new blood vessels (angiogenesis) is essential for a number of physiological and pathophysiological processes such as tissue regeneration, tumor development and the integration of artificial tissues. There are currently no time-lapsed *in vivo* imaging techniques providing information on the vascular network at the capillary level in a non-destructive, three-dimensional and high-resolution fashion. This paper presents a novel imaging framework based on contrast enhanced micro-computed tomography (micro-CT) for hierarchical *in vivo* quantification of blood vessels in mice, ranging from largest to smallest structures. The framework combines for the first time a standard morphometric approach with densitometric analysis. Validation tests showed that the method is precise and robust. Furthermore, the framework is sensitive in detecting different perfusion levels after the implementation of a murine ischemia-reperfusion model. Correlation with both histological data and micro-CT analysis of vascular corrosion casts confirmed accuracy of the method. The newly developed time-lapsed imaging approach shows high potential for *in vivo* monitoring of a number of different physiological and pathological conditions in angiogenesis and vascular development.

## Introduction

*In vivo* analysis of the remodeling process of vascular networks at all hierarchical levels is a powerful way to assess the viability and functionality of native and engineered biological tissues. Changes in the vasculature occur in several physiological and pathological situations and are governed at the micro-vascular level by angiogenesis. Neovascularization or angiogenesis is the formation and growth of new blood capillaries by cellular outgrowth from pre-existing micro-vessels and is a fundamental process in vertebrates. It takes place naturally during development and allows an adequate blood perfusion during the morphogenesis of tissues and organs [1] and the tissue regeneration processes [2], where the re-growing vessels bring the necessary nutrients to the injured tissue. Angiogenesis is also involved in a number of pathological conditions such as psoriasis, rheumatoid arthritis, congestive heart failure, atherosclerosis, peripheral artery disease, and tumor growth. In the latter, tumor progression is usually coupled with the growth of aberrant vessel structures [3]. In recent years, neovascularization has gained attention as a major player in the successful integration of tissue engineered implants. To ensure the proper and fast integration of an implanted artificial tissue, as well as the survival of the tissue itself when seeded with cells, formation and growth of blood vessels have to take place inside the biomaterial [4]. Several strategies have been developed to inhibit (in the case of tumors) or induce (in regenerative medicine) the formation of blood vessels. They target different pro- or anti-angiogenic factors, among them the vascular endothelial growth factor (VEGF), different matrix metalloproteinases (MMPs) and cell-adhesion integrins [5], [6].

The study of vascular formation and development requires a complete three-dimensional *in vivo* analysis of the newly formed vascular network at high resolution and in a time-lapsed fashion. Current methods for *in vivo* vascular imaging present major limitations in the assessment of angiogenesis, especially in small animals such as rats and mice. Magnetic resonance angiography and positron emission tomography are sensitive enough to track angiogenesis but do not provide sufficient resolution for quantitative analysis of the micro-vascular bed [5], [7], [8]. Doppler ultrasound offers increased spatial resolution to monitor blood flow, allowing vascular imaging of conditions such as inflammation and angiogenesis. However, ultrasound images lack anatomical information and therefore require experienced operators for interpretation [9]. Recently, other *in vivo* techniques such as optical imaging have been investigated with increased interest for the assessment of vasculature *in vivo*. However, the major drawbacks of optical approaches include low resolution and low penetration depth [10]. Higher resolution techniques such as histological analysis and micro-computed tomography (micro-CT) of vascular corrosion casts provide a detailed assessment of the vascular network to the micro-vascular level, but cannot be applied *in vivo* for longitudinal monitoring [11]–[14].

*In vivo* micro-CT imaging of small animals such as mice presents unique opportunities to image three-dimensional live processes in a time-lapsed fashion. Due to its high resolution and the high penetration power of X-rays in hard tissues, micro-CT has mainly been applied to image bone and its micro-structure [15], [16]. Imaging of blood vessels with micro-CT requires the enhancement of X-ray absorption with an intravascular contrast agent. Standard approaches for the quantification of blood vessels with micro-CT are based on morphometric analysis, which relies on the evaluation of segmented vessels [17], [18]. However, this approach is limited by the resolution of micro-CT scanners, and does not provide any information about small blood vessels below the resolution of the imaging system (e.g. capillaries). Another approach consists in the evaluation of density [19]. In density analysis, masks of areas of interest are applied to quantify the average X-ray absorption in a time-lapsed fashion. This approach has been used in dynamic imaging essentially to determine the kinetics of contrast agents [20], [21], but has never been applied to quantify the density of small vessels at high resolution.

This paper presents a combined framework for time-lapsed vascular imaging and analysis using micro-CT in an in vivo mouse model of angiogenesis and vascular development. It uses a vascular contrast agent that has recently been shown to be safe and to provide stable intravascular contrast in mice [22]. The method integrates for the first time a traditional morphometric approach for the quantification of large and medium-sized vessels with a densitometric analysis scheme for the quantitative assessment of micro-vasculature. The method was validated by testing its reproducibility, robustness, sensitivity and accuracy showing high potential for *in vivo* monitoring of a number of different physiological and pathological conditions with angiogenesis and vascular development.

## Materials and Methods

### Animal Preparation

Thirty-eight female C57BL/6 mice, 11–15 weeks old (Janvier SAS, Le Genest-St-Isle, France) were housed in an environmentally controlled room at a 12 hour light/dark cycle, with free access to a standard diet and tap water. One week of settling was allowed before starting all procedures. A low-density polyethylene catheter for i.v. injection (0.28/0.64 mm internal/external diameter, Scientific Commodities Inc., USA) was inserted in one of the lateral caudal veins (*V. caudalis*) to provide vascular access and was fixed with tape. Prior to scanning, animals were transferred to a custom-made mouse holder specifically designed for vascular imaging with micro-CT. A bolus (150 µL per 25 g mouse) of ExiTron™ nano 12000 (Miltenyi Biotech, Germany) was injected through this catheter following a pre-contrast scan. All procedures were performed under isoflurane anesthesia (5% for induction and 2% for maintenance, delivered in 0.4 L/min oxygen, through a nose mask, Provet Medical AG, Lyssach, Switzerland). The animals were divided in 4 groups to assess reproducibility (n = 7), robustness (n = 15), sensitivity (n = 16) and accuracy (n = 8 for histological analysis and n = 9 for vascular corrosion casting from the animals previously investigated *in vivo*). All experiments were carried out after receiving the local ethics committee approval (Kantonales Veterinäramt Zürich, Zürich, Switzerland).

### Reproducibility

**Figure 1:**
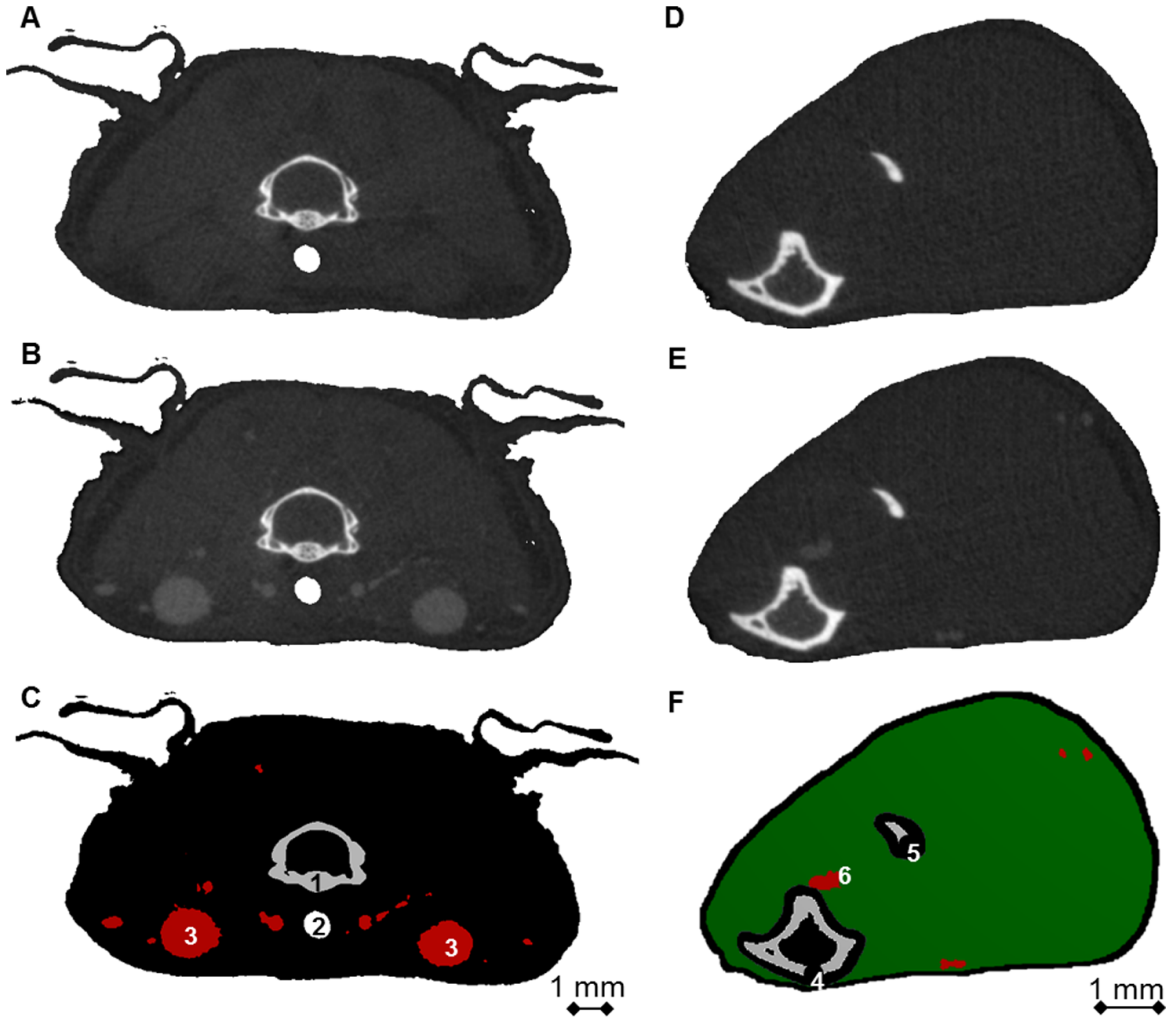
Cross-sections used in the calculation of vascular density. Representative cross-sections of the reference region (neck, A–C) and the region of interest (right lower hind limb, D–F). A) Neck region pre-contrast injection. B) Neck region post-contrast injection. C) In red, mask of the region used to quantify the average X-ray absorption in blood (µ_blood_). D) Lower hind limb region pre-contrast injection. E) Lower hind limb post-contrast injection. F) In green, mask of the region used to quantify the average X-ray absorption in muscle (µ_muscle_). Grey (bone) and black (skin and surroundings of bone) areas were excluded from the evaluation. Numbers represent anatomical structures of interest: 1) Vertebra; 2) Trachea; 3) *V. jugularis*; 4) Tibia; 5) Fibula; 6) *V. poplitea*.

A reproducibility study was carried out on n = 7 animals, which were scanned 5 times each on consecutive days with the following protocol. Pre- and post-contrast scans were acquired at a reference region (neck) and at a region of interest (right lower hind limb) with *in vivo* micro-CT (vivaCT 40, Scanco Medical AG, Brüttisellen, Switzerland). Figure 1 shows representative pre- and post-contrast cross-sections of the neck (Figure 1A and 1B) and right lower hind limb (Figure 1D and 1E). The scanned regions were selected from a scout view taken in dorso-ventral direction. The scanner was operated at 45 kVp and 150 µA, with an integration time of 200 ms, and no frame averaging. 1000 projections were captured over 180 degrees with a field of view of 35 mm in diameter and a length of 3.69 mm, leading to a nominal isotropic resolution of 17.5 µm. All micro-CT scans were performed at controlled air temperature (T = 30°C±0.5°C). The CT scanner was calibrated weekly for mineral equivalent value, and monthly for determining in-plane spatial resolution.

A 3D registration procedure was used to match the image data belonging to the different days [23]. After three-dimensional digital reconstruction of the scanned projections, a Gaussian filter was applied to the images to reduce noise (sigma 1.2, support 3). Pre-contrast images were translated and rotated to match the corresponding post-contrast images using rigid registration [23]. The vascular network was classified in three groups, depending on the vessels thickness (V.Th): the first group comprised the major vessels of the network (V.Th>500 µm), the second group medium-sized vessels (100<V.Th<500 µm), and the third group small vessels (V.Th<100 µm). Each group of vessels was then analyzed with a different image processing protocol. Large vessels of the network (V.Th>500 µm) were segmented from the neck images by a fixed thresholding procedure (16% of the maximum grayscale value). Medium-sized vessels (100<V.Th<500 µm) were segmented from lower hind limb images by a fixed thresholding procedure (16% of the maximum grayscale value) and additional component labeling. A morphological closing procedure (3 dilation and 3 erosion steps) was applied to refill hollow structures that appeared in the vessels after thresholding. Global standard morphometric analysis was performed on large and medium-sized vessels using the software package IPL (Image Processing Language, Scanco Medical AG, Brüttisellen, Switzerland). This software was originally developed to analyze micro-CT data derived from bone biopsies [24]. Assessed parameters included vessel volume fraction (VV/TV), vessel thickness (V.Th), vessel separation (V.Sp) and vessel number (V.N). Detailed descriptions of the morphometric parameters and their calculation are given elsewhere [11]. Morphometric analysis was repeated on the segmented vessels on each consecutive day.

To assess small vessels (V.Th<100 µm), a image processing protocol based on a densitometric analysis was developed. A mask of the muscular tissue that included only small vessels in the lower hind limb (represented in green in Figure 1F) was created by excluding medium-sized vessels previously segmented. This mask was then used to calculate the average X-ray absorption value in the muscular region with small vessels both pre- (µ_pre-muscle_) and post-contrast (µ_post-muscle_). Similarly, the large blood vessels previously segmented in the neck (represented in red in Figure 1C) were used to calculate the average X-ray absorption in blood both pre- (µ_pre-blood_) and post-contrast (µ_post-blood_). The vascular density in the region with small vessels (V.D) was calculated using the following formula:
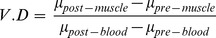



Vascular parameters derived from morphometric and densitometric analysis were quantified for each day and the corresponding precision errors (PEs), intraclass correlation coefficients (ICCs) and relative confidence intervals were calculated according to Glüer et al [25].

### Robustness

A second analysis was performed to assess the influence of the injected dose of contrast agent. The independence of the assessed vascular parameters from the injected volume of contrast material was considered as proof of robustness of the method. The animals were divided into three groups of n = 5 mice each, which received 0%, 50% and 100% of the standard injection dose of contrast agent. Pre- and post-contrast scans were acquired, and morphometric and densitometric analysis were applied in the lower hind limb of the reconstructed images, as previously described for the reproducibility analysis.

### Sensitivity

An established model for angiogenesis was adopted for n = 12 mice to test the sensitivity of the method. An ischemia-reperfusion model was implemented in the right lower hind limb to create different blood perfusion states, as described by Scholz et al [26]. Briefly, the right femoral artery was ligated proximal to the origin of the *arteria poplitea*. This occlusion was expected to cause an immediate reduction of blood flow in the region downstream of the ligation. Subsequently, low levels of oxygen (hypoxia) provoked by low perfusion levels were expected to induce the formation of new blood vessels in the days following the surgery, which would eventually recover the initial perfusion levels of the ligated paw. An additional group of n = 4 animals was sham-operated (only an incision of the skin was made). To assess changes in vascular volume, mice were monitored with micro-CT before, immediately after, and 2 days after surgery with the same scanning protocol described for the reproducibility analysis. Vascular parameters were calculated for medium and small vessels (no large vessels are present in the lower hind limb) with standard morphometry and the previously described densitometric approach, respectively. Laser Doppler imaging was used to measure blood flow levels to ensure consistency of ligation on n = 2 animals, which were sacrificed immediately after the measurements.

### Accuracy: Histological Analysis

Histological sectioning and analysis were carried out on n = 4 animals that underwent the ligation procedure and on n = 4 sham-operated animals. 10 µm thick cryosections of right (ligated) and left (control) lower hind limbs were prepared. The histochemical protocol was carried out similarly to Rosenblatt et al [27]. Briefly, the sections were prepared at −22°C, fixed with 4% paraformaldehyde and rinsed 3 times in phosphate buffered saline (PBS). Following fixation, the slides were incubated for 1 hour at 37°C in a shaker bath. The incubation medium was prepared just prior to use as follows: 300 mg of gelatin was dissolved in 20 mL of 0.1 M tris(hydroxymethyl)aminomethane maleate buffer. 25 mg of adenosine triphosphate (ATP, crystalline disodium salt, Sigma-Aldrich, USA), 3 mL of 0.06 M lead nitrate, 5 mL of 0.068 M calcium chloride, and 20 mL of double-distilled water. The sections were then rinsed 15 times with distilled water. The stain was developed in 2.0% ammonium sulfide for 1 min, after which the sections were washed 3 times in distilled water. All cryosections were coverslipped and viewed using conventional light microscopy. Images were then analyzed with the software package *Fiji Is Just ImageJ* [28]. Blood vessels were segmented by a thresholding procedure and two-dimensional vascular density was calculated for each section.

### Accuracy: Vascular Corrosion Casts

Vascular corrosion casts were produced for *ex vivo* analysis using ultra-high resolution micro-CT. The protocol was adapted from the one described by Beckmann et al [29]. Briefly, n = 9 mice were deeply anesthetized with isoflurane (5%) and pentobarbital (Provet Medical AG, Lyssach, Switzerland). The thorax was opened to expose the heart and aortic arc. A small catheter (Fine Bore Polythene Tubing, 0.58/0.96 mm ID/OD, Smiths Medical, UK) was carefully introduced in the aorta and fixed there with surgical thread (Safil®, B-Braun, Germany). The animals were then perfused through the aorta, first with PBS containing 2% heparin, then with 2% paraformaldehyde in PBS and the polymer resin PU4ii (Polyurethane for Improved Imaging, VasQtec, Switzerland) [30], all infused at 4 mL/min, 100 mm Hg and at body temperature (37°C). The resin is a mixture of PU, solvent (50% ethylmethylketone; EMK) and a blue dye. After resin curing, the lower hind limbs were excised from the animals and the skin was removed. Soft tissue was macerated in 7.5% potassium hydroxide (KOH) for 24 h at 50°C. Bone tissue was kept in the samples as a reference feature to allow future registration with the corresponding *in vivo* image datasets. Casts were then washed with water and stained for 24 h with osmium solution (2% in water) to obtain radiopacity. The samples were then immersed in home-made pluronic gel (polyoxyethylene-polyoxypropylene triblock copolymer), a thermo-reversible gel that is solid at room temperature and liquid below. The prepared samples were scanned with micro-CT (µCT 50, Scanco Medical AG, Brüttisellen, Switzerland). The scanner was operated at 55 kVp and 145 µA, with an integration time of 200 ms, and frame averaging of 3. All micro-CT scans were performed at room temperature. The CT scanner was calibrated weekly for mineral equivalent value, and monthly for determining in plane spatial resolution. An imaging protocol consisting of two consecutive scans was carried out to allow the identification of the same region previously scanned *in vivo*. The first scan was acquired at medium resolution to provide an overview for orientation within the sample; the second one at ultra-high resolution for detailed vascular analysis of the matching region previously investigated *in vivo*. In the medium-resolution scan, the following settings were applied: 2000 projections were captured over 180 degrees with a field of view of 10 mm in diameter and a total length of 3.70 mm, leading to a nominal isotropic resolution of 10 µm. The medium-resolution images were then downscaled to 17.5 µm to match the *in vivo* voxel size and the two image datasets (*in vivo* and *ex vivo*) were registered using rigid registration [23]. After identification of the matching region, an ultra-high resolution scan was carried out at the same position with the following settings: 2000 projections were captured per 180 degrees with a field of view of 7 mm in diameter and a total length of 1.86 mm, leading to a nominal isotropic resolution of 1.4 µm. A Gaussian filter was applied to the ultra-high resolution images to reduce noise (sigma 1.2, support 1). Blood vessels were segmented by a fixed thresholding procedure (20% of the maximum grayscale value) and bone was excluded by manual contouring. A morphological closing procedure developed in house using the software package IPL (Image Processing Language, Scanco Medical AG, Brüttisellen, Switzerland) was applied and used to close holes that could have appeared after thresholding. Global standard morphometric analysis was performed on the ultra-high resolution images again using the software package.

Statistical analysis was performed with the software package R (R: A Language and Environment for Statistical Computing) [31]. An unpaired Student’s t-test was used to compare the vascular parameters over the monitored days. For multiple comparisons, the significance of differences was evaluated using analysis of variance (ANOVA) followed by the Bonferroni test. An analysis of the differences between the techniques was done according to the method described by J. M. Bland and D.G. Altman [32]. P values smaller than 0.05 were considered significant. All data are shown as mean ± standard deviation.

## Results

### Reproducibility

Table 1, 2 and 3 report the precision errors (PEs), the intraclass correlation coefficients (ICCs) and relative confidence intervals for morphometric and densitometric analysis for large, medium and small vessels. Precision errors in morphometric analysis for large vessels ranged between 5.09% and 8.27%. These errors increased when analyzing medium-sized vessels, for which PEs ranged between 9.47% and 12.56%. The standard morphometric analysis of medium vessels presented lower errors than the density-based approach (PE = 13.87%). Small vessels could be assessed only with densitometric analysis, with a precision error of 11.92%. Extremely low precision errors (PE = 0.92%, 0.94%) were found when analyzing the average X-ray absorption in muscle (µ_pre-muscle_ and µ_post-muscle_). Furthermore, in the densitometric approach, although the PEs are relatively large, the ICCs are still similarly high as compared to the morphometric analysis of the medium sized vessels.

**Table 1:** Reproducibility of morphometric analysis for large vessels.

LARGE VESSELS (V.Th>500 µm)						
Morphometric analysis						
Parameters	Mean	PE_SD_	PE_%CV_	CI_95%_PE_%CV_	ICC	CI_95%_ICC_%CV_
**VV/TV (%)**	1.031	0.061	5.92%	3.99–6.27%	0.901	0.893–0.907
**VS/TV (1/mm)**	0.071	0.005	7.31%	6.15–8.57%	0.852	0.848–0.854
**V.Th (mm)**	0.760	0.058	7.57%	6.20–8.58%	0.887	0.880–0.894
**V.Sp (mm)**	1.607	0.133	8.27%	6.22–9.14%	0.878	0.866–0.883
**V.N (1/mm)**	0.824	0.042	5.09%	4.30–7.78%	0.902	0.900–0.905

PE = Precision Error; ICC = Intraclass Correlation Coefficient. Assessed parameters are: VV/TV (Vessel Volume per Tissue Volume), VS/TV (Vessel Surface per Tissue Volume), V.Th (Vessel Thickness), V.Sp (Vessel Spacing), V.N (Vessel Number). N = 7.

**Table 2:** Reproducibility of morphometric and densitometric analysis for medium vessels.

MEDIUM VESSELS (100<V.Th<500 µm)						
Morphometric analysis						
Parameters	Mean	PE_SD_	PE_%CV_	CI_95%_PE_%CV_	ICC	CI_95%_ICC_%CV_
**VV/TV (%)**	0.457	0.046	9.98%	8.54–11.27%	0.802	0.800–0.803
**VS/TV (1/mm)**	0.259	0.027	10.32%	7.87–12.42%	0.792	0.790–0.795
**V.Th (mm)**	0.105	0.013	12.56%	10.38–15.32%	0.788	0.786–0.802
**V.Sp (mm)**	1.028	0.097	9.47%	8.88–11.22%	0.801	0.800–0.803
**V.N (1/mm)**	1.248	0.141	11.27%	9.79–13.44%	0.795	0.790–0.800
**Densitometric analysis**						
**V.D (%)**	0.382	0.052	13.87%	10.88–15.92%	0.799	0.792–0.805

PE = Precision Error; ICC = Intraclass Correlation Coefficient. Assessed parameters are the same as those calculated for large vessels. N = 7.

**Table 3:** Reproducibility of densitometric analysis for medium vessels.

SMALL VESSELS (V.Th<100 µm)						
Densitometric analysis						
Parameters	Mean	PE_SD_	PE_%CV_	CI_95%_PE_%CV_	ICC	CI_95%_ICC_%CV_
**V.D (%)**	2.121	0.253	11.92%	9.95–13.02%	0.820	0.818–0.827
**µ_pre-blood_ (1/mm)**	0.903	0.019	2.06%	1.88–2.56%	0.850	0.848–0.856
**µ_post-blood_ (1/mm)**	1.575	0.047	3.01%	2.55–3.98%	0.842	0.839–0.850
**µ_pre-muscle_ (1/mm)**	0.923	0.005	0.57%	0.47–0.61%	0.901	0.889–0.905
**µ_post-muscle_ (1/mm)**	0.937	0.006	0.65%	0.61–0.72%	0.893	0.890–0.897

PE = Precision Error; ICC = Intraclass Correlation Coefficient. Assessed parameters are: V.D (Vessel Density), µ_pre-blood_ (average X-ray absorption in blood pre-contrast injection), µ_post-blood_ (average X-ray absorption in blood post-contrast injection), µ_pre-muscle_ (average X-ray absorption in muscle pre-contrast injection), and µ_post-muscle_ (average X-ray absorption in muscle post-contrast injection). N = 7. µ_pre-blood_.

### Robustness

**Figure 2:**
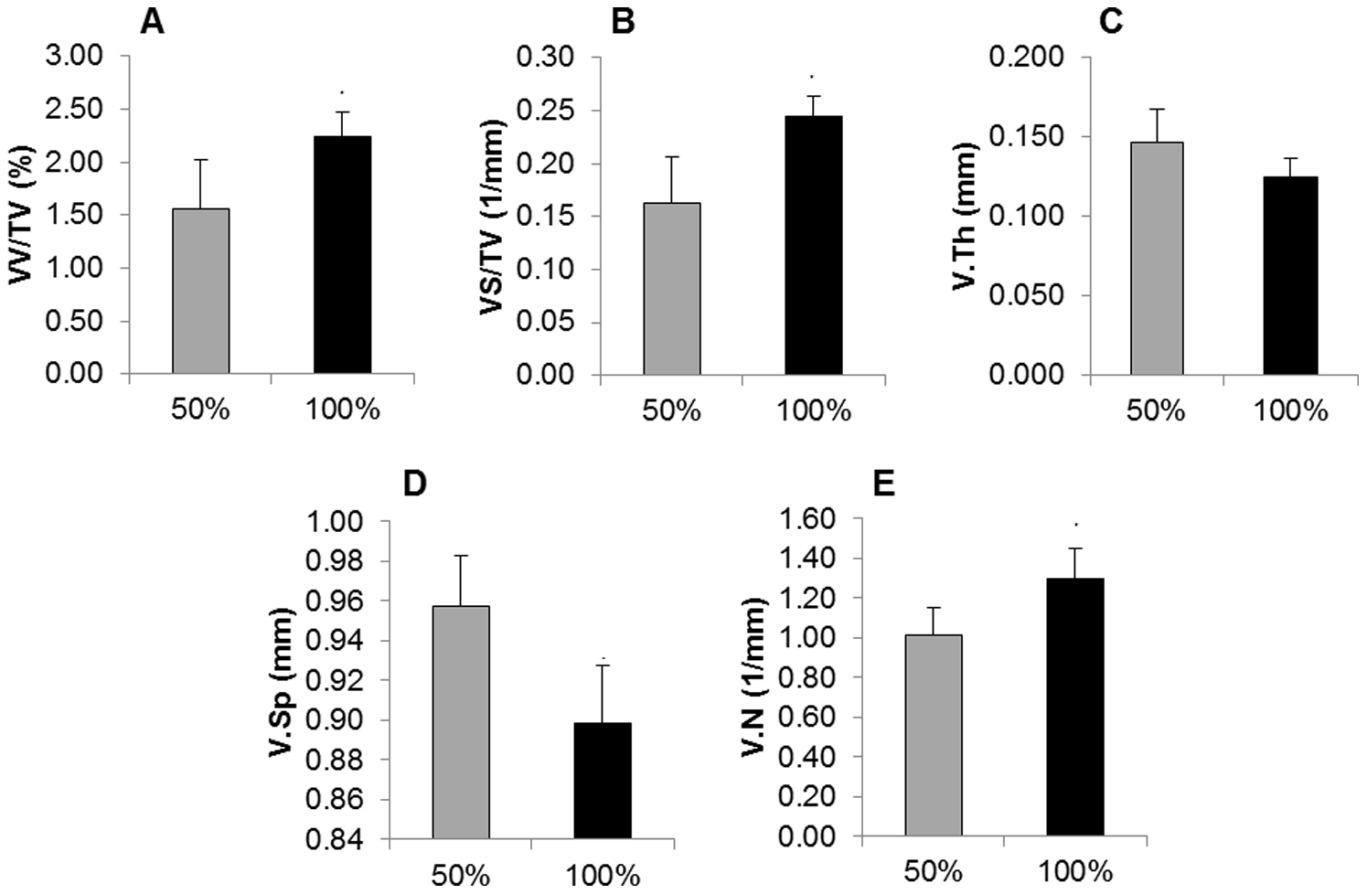
Robustness results for morphometric analysis of the lower hind limb when 50% and 100% of the standard contrast volume were injected. All assessed parameters (A) VV/TV, B) VS/TV, C) V.Th, D) V.Sp, E) V.N) exhibited significant differences between half and full dose measurements. ***p<0.001, **p<0.01. N = 7/group.

**Figure 3:**
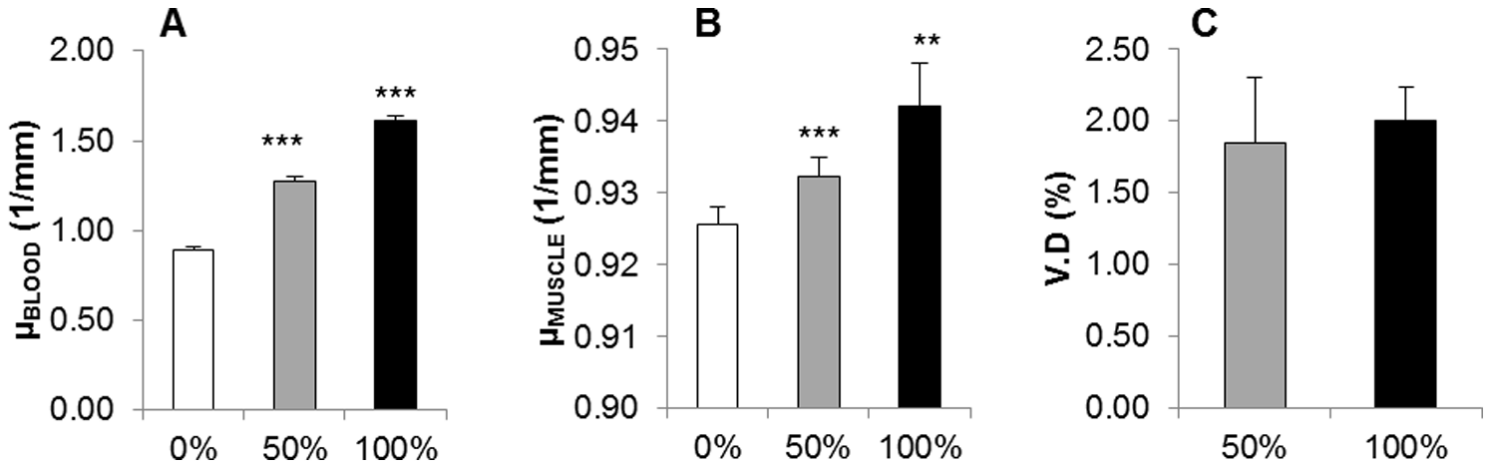
Robustness results for densitometric analysis of the lower hind limb. Average X-ray absorption values in blood (A) and muscle (B) when 0%, 50% and 100% of the standard contrast volume were injected. Significant differences were found between all three cases. C) Vascular density values calculated with the densitometric analysis corresponding to 50% and 100% of the standard contrast volume. No significant differences were found for vascular density, which indicated that it is not influenced by the injected dose of contrast agent. ***p<0.001, **p<0.01. N = 7/group.

Figure 2 presents the standard morphometric parameters calculated in the lower hind limb for 50% and 100% of the injected contrast agent dosage. Significant differences between the two doses were found for all assessed parameters. The densitometric analysis revealed the average X-ray absorption in blood (Figure 3A) and in the in muscular tissue with small vessels (Figure 3B) and the corresponding values for vascular density (Figure 3C). Significant differences in X-ray absorptions were found between 0% and 50% and between 50% and 100% of the injected dose for both blood and muscle. No significant differences were found for vascular density values calculated from the 50% or 100% dose measurement, although higher standard deviations were found in the 50% case.

### Sensitivity

**Figure 4:**
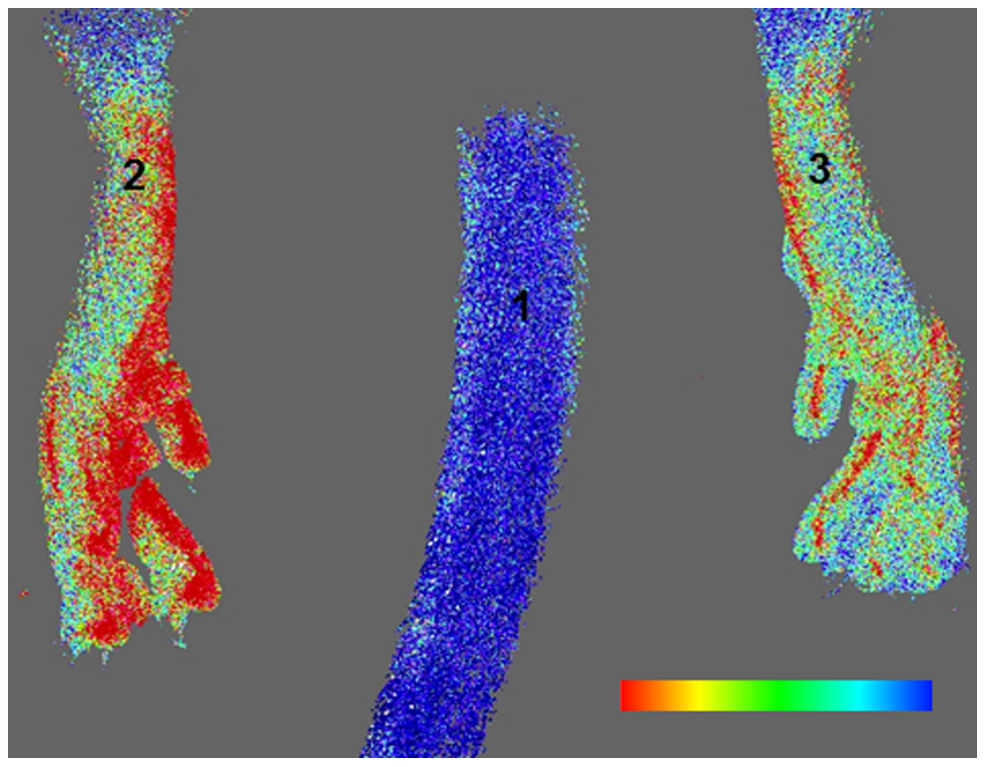
Blood perfusion map of the mouse lower hind limbs 2 days after ligation of right femoral artery, obtained with laser Doppler imaging. The color bar represents blood flow (or perfusion) levels, from blue (low) to red (high). Note the much lower perfusion levels of the right (ligated) paw compared to the left (control) paw.

**Figure 5:**
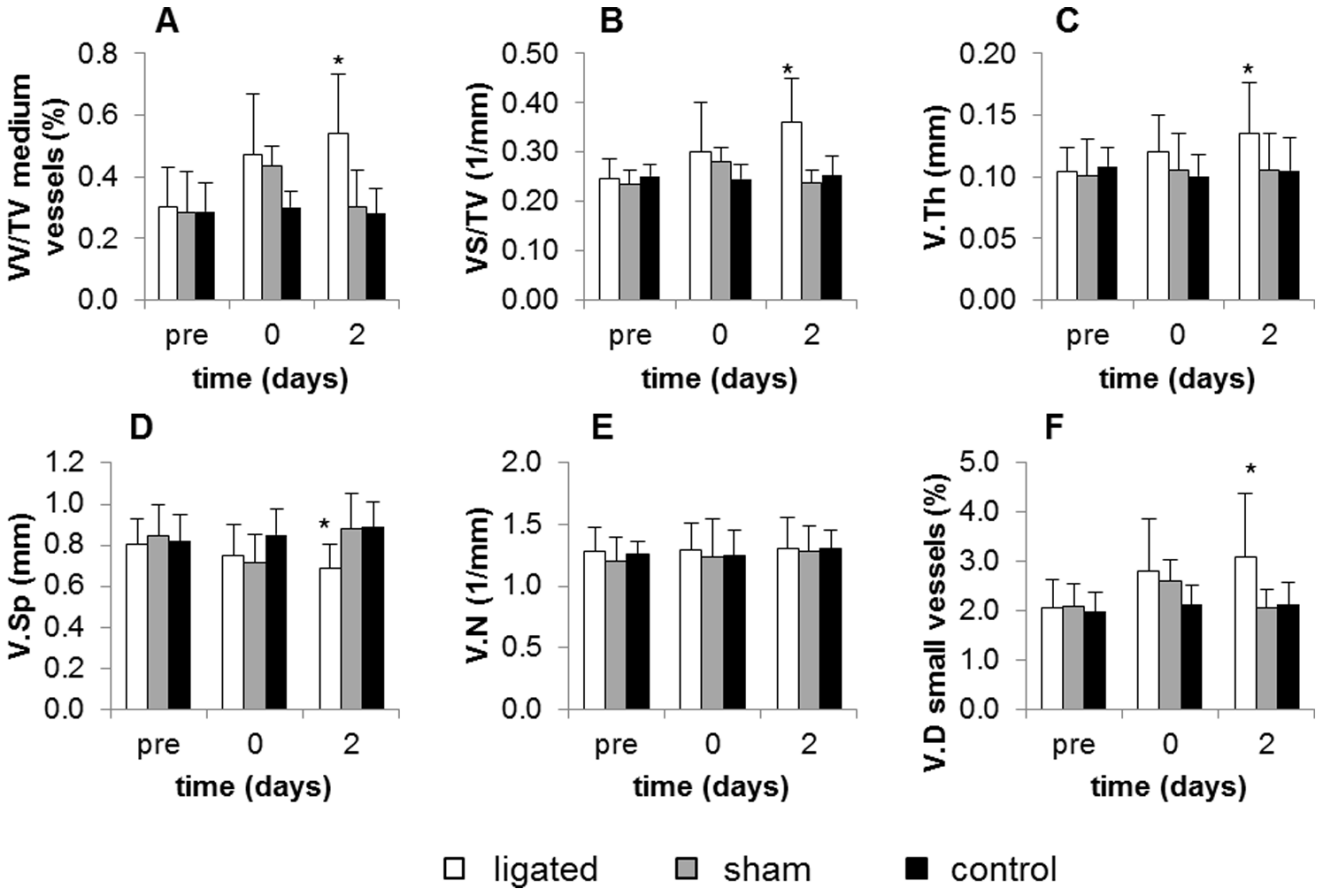
Time course of vascular parameters. A–E represent parameters calculated for medium vessels from morphometric analysis, while F is calculated for small vessels from densitometric analysis. In morphometric analysis, VV/TV of medium vessels, VS/TV, V.Th, and V.Sp of ligated paws exhibited significant differences on day 2 with respect to pre-surgery sham and control values. In the densitometric analysis, V.D of small vessels of ligated paws also exhibited a significant difference with respect to pre-surgery sham and control values. *p<0.05. N = 12 for ligated group, N = 4 for sham-operated group, and N = 7 for control group (from reproducibility study).

Blood perfusion maps of lower hind limbs were obtained with laser Doppler imaging at 2 days after ligation. Figure 4 shows that the right (ligated) paw presented much lower perfusion levels compared to the left (control) paw, which confirmed successful arterial occlusion. Figure 5 presents the time-course of vascular parameters obtained from standard morphometric analysis for medium-sized vessels (Figure 5A–E) and from densitometric analysis for small vessels (Figure 5F). The morphometric analysis showed that medium-sized vessels of ligated paws significantly increased their average volume, surface, and thickness, with respect to the corresponding pre-surgery, sham, and control values. A significant decrease in vessel spacing was also observed (Figure 5D). Significant differences were found only at the small vessel level with the densitometric analysis: an increase in vascular density was noticed after surgery, which became significant as early as 2 days after arterial occlusion, with respect to the pre-surgery levels, indicating new vessel formation.

### Accuracy: Histological Analysis

**Figure 6:**
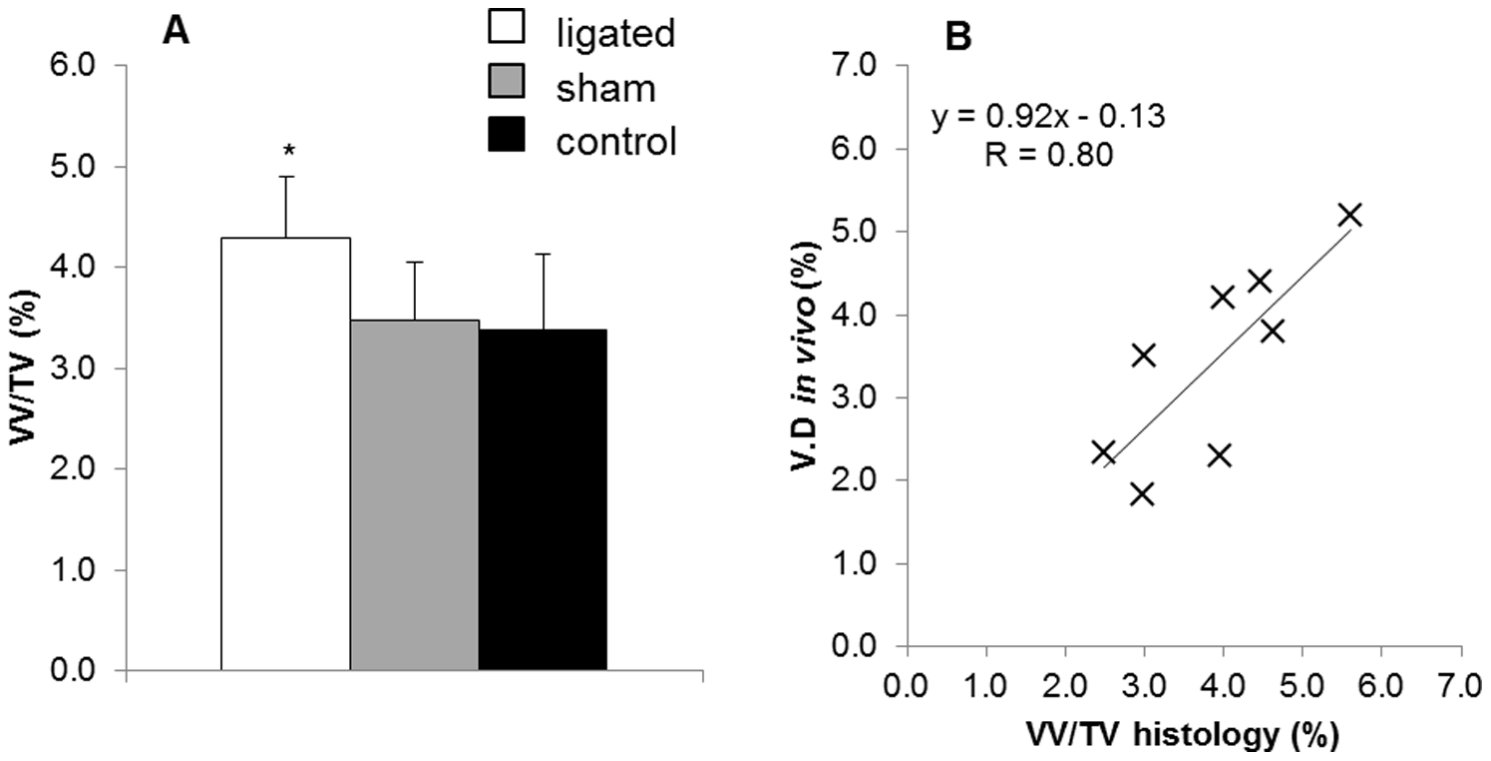
Results obtained from histological analysis. A) Vascular density calculated with histological analysis for ligated, sham-operated and control paws. Significant differences were found for ligated paws as compared to the other groups. B) Correlation curve for VV/TV and V.D using histological analysis as reference imaging technique. N = 4/group.

The analysis of the histological sections showed higher vascular density values in the ligated paws versus the non-ligated (control) and the sham-operated paws (Figure 6A). Correlation between vascular densities previously calculated *in vivo* with the densitometric analysis and vascular densities quantified from histological sections resulted in an R value of 0.80 and a slope of 0.92 (Figure 6B, p<0.01). The analysis of the differences between the *in vivo* technique and the histological analysis using the Bland-Altman test revealed an R value of 0.26 (p = 0.52).

### Accuracy: Vascular Corrosion Casts

**Figure 7:**
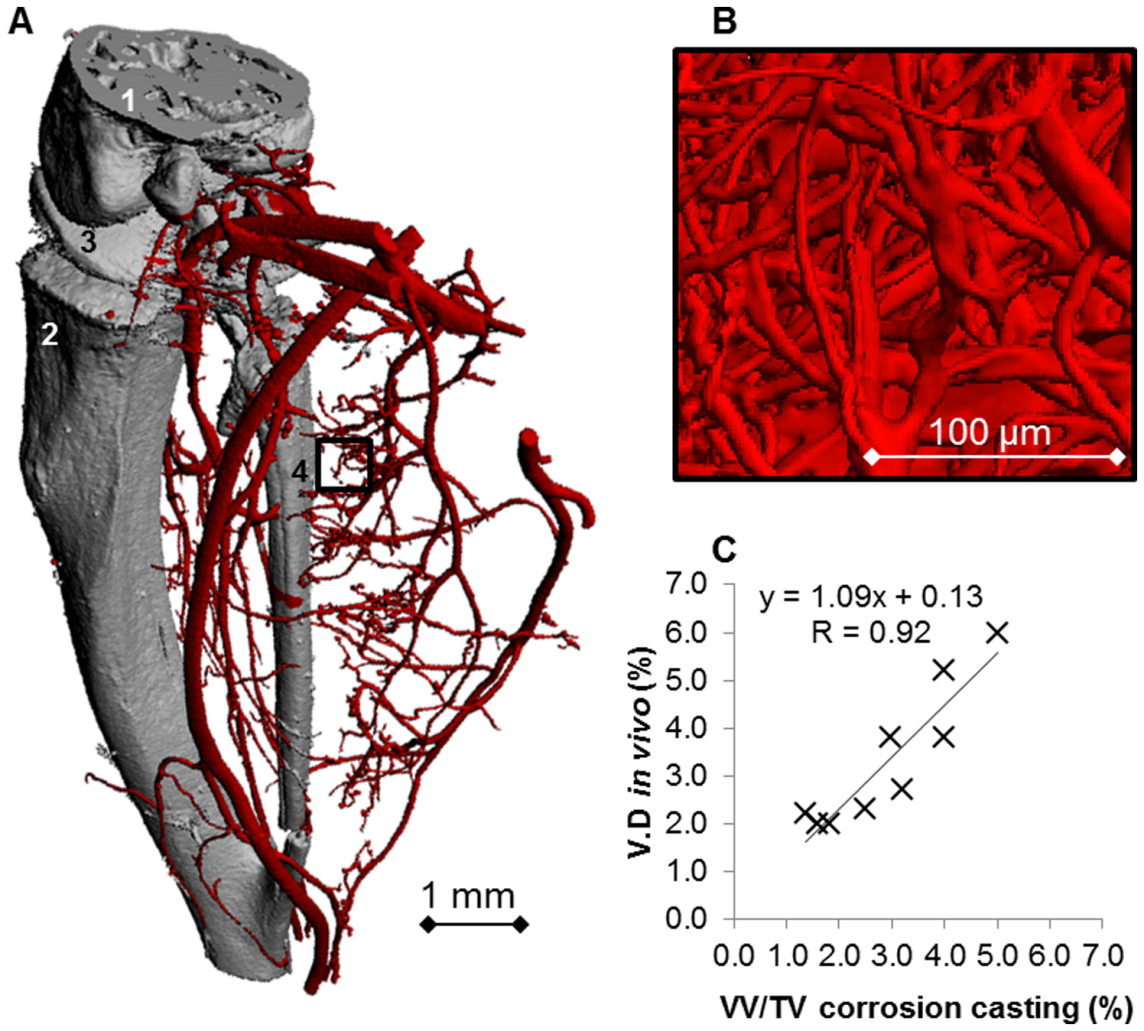
Results obtained from vascular corrosion cast analysis. A) Overview of a vascular corrosion cast (without corrosion of hard tissues) of the right lower hind limb, scanned with medium-resolution (10 µm) micro-CT. Blood vessels are represented in red, bone in grey. Numbers represent anatomical structures of interest: 1) Femur; 2) Tibia; 3) Knee joint; 4) Fibula. B) Detail of the lower hind limb vasculature obtained by micro-CT scanning at ultra-high resolution (1.4 µm). Notice that the capillary network can be clearly visualized. C) Correlation curve for VV/TV and V.D using ultra-high resolution micro-CT of vascular corrosion casts as a reference imaging technique.

Medium-resolution (10 µm) micro-CT of vascular corrosion casts allowed the three-dimensional visualization of the large and medium-sized vascular network in the lower hind limb (Figure 7A). This overview was used for sample orientation but was not sufficient to represent the micro-vascular network. Instead, ultra-high resolution (1.4 µm) micro-CT scans allowed the visualization of the vasculature down to the smallest vessels (the capillary bed, represented in Figure 7B). Correlation between vascular densities previously calculated *in vivo* with the densitometric analysis and vascular densities quantified from ultra-high resolution micro-CT images resulted in an R value of 0.92 and a slope of 1.09 (Figure 7C, p<0.01). The analysis of the differences between the *in vivo* technique and the corrosion casting analysis using the Bland-Altman test revealed an R value of 0.40 (p = 0.29).

## Discussion

*In vivo* investigation of the vascular network at all hierarchical levels requires an appropriate non-invasive, high-resolution imaging technique. Morphometry is a standard approach for vascular analysis that can be applied when blood vessels are segmentable from the surrounding soft tissues. Therefore, its applicability depends mostly on the resolution provided by the *in vivo* micro-CT scanner and by the vascular contrast agent used. As most of the *in vivo* micro-CT scanners can visualize vessels typically down to 10–15 µm, morphometry can be employed for quantifying medium to large vessels, but it cannot be used to analyze small vessels that are below the resolution of the imaging system. The quantification of small vessels is essential as changes in the network, which later reflect on all the hierarchical structure of blood vessels, start at the micro-vascular level. Furthermore, our reproducibility analysis proved that although morphometry is a reproducible technique for large vessels, its repeatability diminishes when the size of the analyzed vessels is decreased. Vascular analysis based on assessment of the density of the contrast agent present in the vessels is an imaging approach that was used here for the first time for the quantification of small vascular structures, in combination with standard morphometric analysis. It is based on the evaluation of the change in grey values after contrast injection and it requires a reference scan for the calibration of contrast agent concentration in blood. In addition, it necessitates the use of a vascular contrast agent that provides stable concentration levels in the blood during the entire scan (about 30 minutes in total) and no extravasation of contrast from the intravascular compartment. These requirements have been found true for ExiTron™ nano 12000 in previous work [33]. In contrast to standard morphometric analysis, the reproducibility of densitometric analysis decreases when the size of the analyzed vessels is increased. Therefore, this method is more precise when analyzing a region with only small vessels representing a relatively homogenous area. Details of the grayscale values used to calculate the vascular density suggest that errors are mostly due to the choice of the reference region, in our study the neck. Unfortunately, this region suffers from movement artifacts due to breathing and heart beating. Therefore, better results in terms of precision could be obtained with respiratory/cardiac gating, although this significantly lengthens the scanning time.

The results revealed by the robustness study showed that the densitometric analysis of vascular density levels is independent from the contrast agent dose, as the X-ray absorption values of the lower hind limb are calibrated with the absorption values in blood analysed in the reference scan. The morphometric approach, however, is not a calibrated analysis, as it is strongly influenced by the tracer dose. In this case, the injected volume of contrast agent determines the dimensions of the smallest visible vessels (defined as the resolution of the contrast agent), and therefore the corresponding morphometric parameters.

In our sensitivity study, the expected initial dramatic decrease in blood flow appeared in the regions downstream of the ligation, which was confirmed by laser Doppler imaging 2 days after surgery (Figure 4). Numerous studies in the literature attest that this loss in blood flow, which happens immediately after ligation, is followed by a slow recovery of the original perfusion levels [14], [18], [26]. Parallel to these changes in perfusion, an increase in vascular volume caused by angiogenesis and arteriogenesis of collateral vessels is expected a few days after ligation. Our results showed that, *in vivo,* only the newly developed densitometric approach was sensitive enough to detect an increase in the volume of small blood vessels as early as 2 days after ligation (Figure 5F). Standard morphometric analysis was also applied to analyze medium-sized vessels, and revealed an increase in medium-sized vessel volume, surface, and thickness (Figure 5A–C). This increase is the result of an enlargement of collateral blood vessels in an effort to compensate for the loss in blood flow caused by the ligation.

Histology and vascular corrosion cast analysis were carried out to determine the accuracy of the method and their vascular densities were quantified and compared to the values found with the densitometric approach. Further parameters such as vessel thickness and spacing can be calculated only with *ex vivo* analyses but they cannot be used for the assessment of the method accuracy. Analysis of stained histological sections is the gold standard technique for characterization of blood capillaries. Sample shrinkage is one of the major drawbacks during the preparation of histological sections. For this reason, tissue fixation was carried out using paraformaldehyde (and not with precipitating fixatives such as alcohols), which cross-links tissue proteins and is known for better preservation of tissue morphology [34]. With this protocol, no major signs of shrinkage were noticed on the prepared samples. Histological analysis confirmed higher vascular density values in the occluded versus the non-occluded (control) paws and showed good correlation with the results previously obtained *in vivo* with the densitometric analysis. Vascular corrosion casts represent a high resolution replica of the whole vascular network of an animal. They have been used to study the vasculature in detail [11], [12], [30]. Micro-CT of corrosion casts showed higher correlation values (R = 0.92) with respect to correlation values with histology (R = 0.80). These results might be related to the fact that corrosion cast analysis is three-dimensional, while histology provides only a two-dimensional evaluation. Higher correlation with corrosion casts might also be related to the new sample preparation protocol of corrosion casts, which was specifically developed to match corresponding regions in *in vivo* and *ex vivo* micro-CT images. In this protocol, bone was not corroded from the sample, as a standard preparation protocol would require. Instead, it was kept within the sample and used as a reference structure for image registration between *in vivo* and *ex vivo* datasets. The Bland-Altman analysis revealed that there is no significant correlation between the means and the differences for both the comparisons with histology and corrosion casting, indicating that the *in vivo* densitometric analysis is not affected by a systematic error. Figure 7B represents the level of detail that can be obtained in the vascular network with ultra-high resolution micro-CT scanning. However, although each single capillary can be visualized, both histology and corrosion casting cannot be used for the *in vivo* monitoring of vascular changes, and can therefore only be applied as an endpoint.

The newly developed integrated framework for vascular analysis proved to be a promising high-resolution imaging method for the *in vivo* three-dimensional assessment of blood vessels. It combines standard morphometric analysis with a densitometric evaluation specifically developed for small vessel quantification. To our knowledge, a systematic analysis of standard morphometry for vascular evaluation is presented here for the first time. Furthermore, no method is currently available that provides assessment of small vessels density in a high-resolution, three-dimensional and non-invasive fashion. This framework can be applied in longitudinal studies to non-destructively monitor vascular structures over time, from major to small vessels. The method can be employed in a number of applications, such as in tissue engineered implants for time-lapsed evaluation of successful tissue regeneration. It can also be applied for monitoring tumor vascularization, although special care must be taken as tumor vessels present higher levels of leakiness. In this case, further analysis needs to be carried out to examine the potential extravasation of the contrast agent from the vascular compartment. A limitation to the densitometric approach is that it is not possible to distinguish between the contribution of an increase in the number or volume of blood vessels, as increases in vascular density could be caused by the formation of new blood vessels (angiogenesis) or by the enlargement of existing vessels. A second limitation is that it cannot be applied to study the vasculature in the organs of the reticulo-endothelial system (where this particular contrast agent accumulates) and in hard tissues, where it is extremely difficult to distinguish between bone and the contrast provided by the vascular agent.

## Conclusions

This paper presents a unique framework for vascular analysis based on standard morphometric evaluation and on a densitometric approach. The combination of these two analyses provides complete information on the vascular network at all hierarchical levels. This time-lapsed imaging approach shows high potential for *in vivo* monitoring of a number of different physiological and pathological conditions with angiogenesis and vascular development.
